# Squamous cell carcinoma with apocrine features of the breast: A case report

**DOI:** 10.3892/ol.2014.1800

**Published:** 2014-01-15

**Authors:** YOSHIKI NAITO, RIN YAMAGUCHI, MASAYA TANAKA, AKIHIRO SARUWATARI, YOSHIKUNI TORII, TAKAHISA TSUKAMOTO, NAOYO NISHIDA, KOICHI HIGAKI

**Affiliations:** 1 Department of Pathology, St. Mary’s Hospital, Kurume 830-8543, Japan; 2 Department of Pathology and Laboratory Medicine, Kurume University Medical Center, Kurume 839-0863, Japan; 3 Department of Surgery, St. Mary’s Hospital, Kurume, Fukuoka 830-8543, Japan; 4 Department of Radiology, St. Mary’s Hospital, Kurume, Fukuoka 830-8543, Japan

**Keywords:** breast, breast cancer, squamous cell carcinoma, apocrine carcinoma

## Abstract

A rare case of squamous cell carcinoma (SCC) with apocrine features was investigated; the focus was on the histological characteristics of the cancer cells in a 68-year-old female exhibiting an ulcerated lesion of the right breast. Diagnostic imaging methods identified a lobulated solid tumor and indicated multiple enlarged lymph nodes in the left axilla, which confirmed the diagnosis of advanced breast cancer; thus, a mastectomy was performed. Macroscopic investigations identified the tumor as a white, solid lesion measuring 66 × 68 × 47 mm, which exhibited necrosis. Histologically, the tumor was predominantly solid and exhibited nest patterns, in addition to intracellular keratinization. Immunohistochemical staining identified the tumor cells as positive for cytokeratin 5/6, 34βE12 and P63. The lesion was considered to be an SCC demonstrating negative expression for the human epidermal growth factor receptor 2, estrogen receptor and progesterone receptor; therefore, the tumor was a triple-negative breast cancer. Conversely, approximately one-third of the tumor cells indicated abundant eosinophilic cytoplasm and gross cystic disease fluid, which was demonstrated via protein-15 staining; this indicated the presence of apocrine features. In addition, the androgen receptor was expressed in the tumor cells, thus the lesion was diagnosed as an SCC of the breast, exhibiting apocrine features.

## Introduction

A number of primary breast cancers arise from the mammary gland; however, occasionally tumors exhibit metaplastic features with a variety of histologic findings (1–8) or apocrine metaplasia which is termed apocrine carcinoma (AC) (9,10). Squamous cell carcinomas (SCCs) of metaplasic breast cancers and ACs contain cancer cells with eosinophilic cytoplasm, which complicates the pathomorphological differentiation, although the tumor grade varies between the two cancer types. Moreover, SCCs are often triple-negative (TN) cancers, negative for the expression of the estrogen receptor (ER), progesterone receptor (PgR) and human epidermal growth factor receptor 2 (HER2) and certain ACs are TN cancers (11). However, the prognosis of AC is favourable (12), whereas the prognosis for SCCs is generally poor (13). In the present report, pathomorphological and immunohistochemical (IHC) data that was obtained from SCC exhibiting apocrine features were analyzed with a focus on the histological characteristics of the cancer cells.

## Case report

### Clinical summary

**Figure 1:**
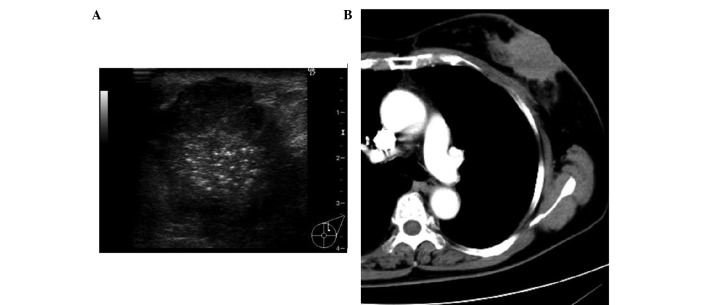
Diagnostic imaging. (A) Ultrasound imaging identified a lobulated, solid tumor measuring ≥3 cm of the left mammary gland. (B) Computed tomography of the chest indicated a mass lesion in the apocrine carcinoma region of the left mammary duct exhibiting heterogeneous and moderately enhanced microcalcifications.

A 68-year-old female attended St. Mary’s Hospital (Kurume, Japan) to undergo examination of an ulcerated lesion of the right breast. Ultrasound imaging demonstrated a lobulated solid tumor ~40×30 mm containing multiple stippled calcifications in the left mammary gland (Fig. 1A). A computed tomography scan displayed a heterogeneously hypodense mass lesion in the left gland and indicated multiple enlarged lymph nodes in the left axilla (Fig. 1B). Magnetic resonance imaging identified a lesion of irregular mass, measuring 66 × 68 × 47 mm in addition to skin infiltration, located in the left breast. Furthermore, a contrast-enhanced dynamic study of the lesion exhibited hyperintensities during the early phase and was marginally washed out in the delayed phase, six min post-contrast. The lesion was therefore considered to be advanced breast cancer and a mastectomy was performed.

### Postoperative period

In the postoperative period, the patient did not consent to receiving chemotherapy; however, the follow-up procedures were continued. A mass lesion resulted from the surgical wound three months postoperatively and was subsequently removed. The pathological findings of the mass were consistent with those of tumor relapse. The patient was informed repeatedly concerning chemotherapy, however, did not provide consent. Eight months following surgery, the patient developed radiographically evident metastasis of the lung and the liver. The patient was informed again regarding chemotherapy and consented to treatment; the patient received epirubicin (75 mg/m^2^), fluorouracil (500 mg/m^2^) and cyclophosphamide (500 mg/m^2^) for four, 21-day cycles. At the time of writing, the patient was receiving the first cycle of docetaxel (60 mg/m^2^), was classified as Performance Status 1 (http://ecog.dfci.harvard.edu/general/perf_stat.html) and was leading an independent life.

### Pathological findings

**Figure 2:**
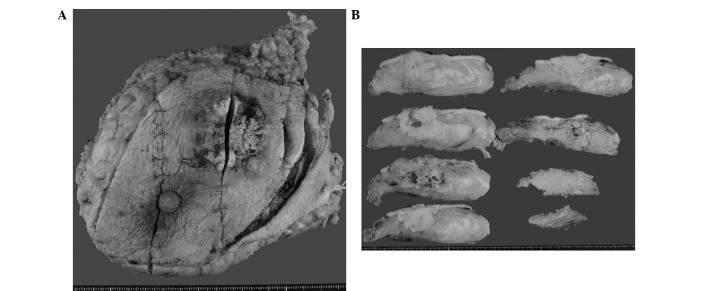
Macroscopic images. (A) The predominant lesion of the tumor was white and solid, measuring 61×27 mm and was associated with a cutaneous ulcer. (B) The tumor exhibited an extensive area of necrosis.

**Figure 3:**
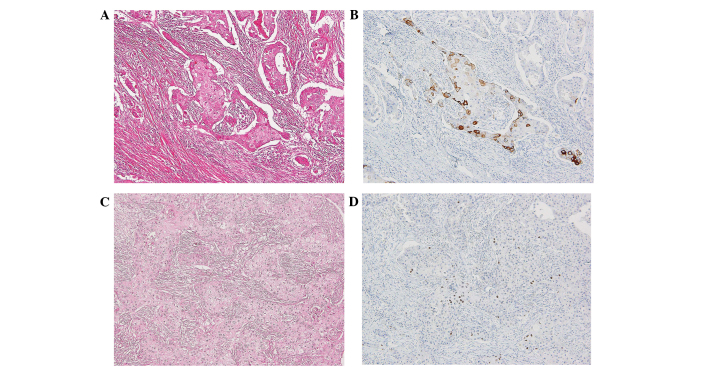
Cancer cells formed nests and exhibited keratinization. (A and C) The histological analysis showed structures comprising of predominantly solid and nest patterns, with keratinization (H&E; magnification, ×100). The tumor cells were positive for (B) cytokeratin 5/6 (magnification, ×100) and (D) P63 (magnification, ×100).

**Figure 4:**
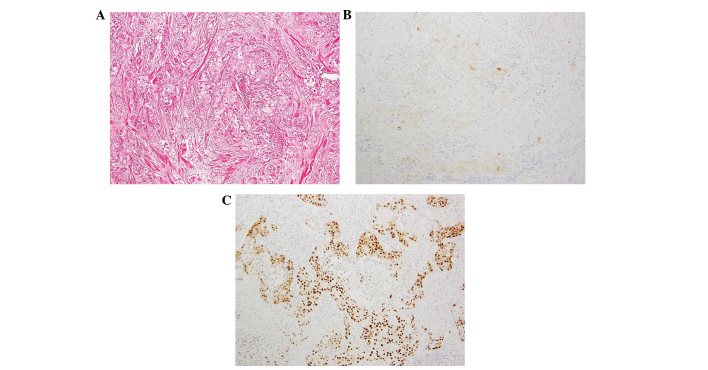
Areas exhibiting apocrine features within the tumor cells. (A) Hematoxylin and eosin staining identified that the areas with apocrine features were abundant in eosinophilic cytoplasm. (B) Gross cystic disease fluid protein-15 was positive in the areas exhibiting apocrine features. (C) The androgen receptor was expressed in the cytoplasm and the nucleus of the tumor cells, which exhibited apocrine features.

Macroscopic investigations identified the lesion as white and solid, measuring 61×27 mm, which exhibited an extensive area of necrosis (Fig. 2A and B). The lesion was identified as neoplastic, via histological analysis, and was predominantly composed of tumor cells arranged in nests with solid regions of varying size and shape. The lesion exhibited moderate glandular lumen formation and comedo-like necrosis (Figs. 3A and 4B), which extended into the surrounding ducts and infiltrated the adipose tissue and skin. The numerous cancer cells that formed such nests exhibited nuclear enlargement, nuclear pleomorphism and an increased nuclear to cytoplasmic ratio, in addition to intracellular keratinization and intercellular bridges. The IHC analysis identified the cancer cells as positive for cytokeratin (CK) 5/6, 34βE12 and P63. The lesion exhibited squamous features (Fig. 3A–D) with a histological grade of three and was negative for the expression of ER, PgR and HER2; the tumor was a triple-negative breast cancer. In contrast to the pathomorphology of pure squamous features, approximately one-third of the tumor area was invaded by cancer cells with abundant eosinophilic cytoplasm and a positive expression for gross cystic disease fluid protein-15 (GCDFP-15). The androgen receptor (AR) was expressed in the cytoplasm and/or the nucleus within the tumor cells (Fig. 4A–C); thus, the tumor appeared to exhibit apocrine features. Based on the histological analysis, the lesion was diagnosed as an SCC of the breast, with apocrine features and documented lymph node metastasis. Written informed consent was obtained from the patient.

## Discussion

SCC is generally characterized by histologically identified keratinization and intercellular bridges (2,14). AC is defined as a carcinoma in which >90% of tumor cells exhibit abundant eosinophilic cytoplasm (15,16). Although histological classification is generally based on the structure and status of a tumor, AC is classified according to the tumor cell findings rather than the tumor structure. In the present case, the cancer cells exhibited abundant eosinophilic cytoplasm containing eosinophilic granules, thus demonstrating apocrine differentiation. These cells, however, did not account for >90% of total cells, thus the findings were considered to indicate apocrine features occurring within an SCC.

The IHC analysis identified that more than half of the squamous cell features expressed high molecular weight keratins (CK14, CK5/6 and 34βE12) and P63 (14). The present case may, therefore, be diagnosed as an SCC due to the presence of keratinization and intercellular bridges, as well as the positive expression of high molecular weight keratin and P63 (Fig. 3A–D). In addition to the SCC, the apocrine features were documented pathomorphologically and using IHC staining (Fig. 4A–C). Generally in AC, a characteristic steroid receptor expression profile defines these tumors as consistently ER-negative, PR-negative and AR-positive (17–19), and previous IHC studies identified that GCDFP-15 was expressed in ~75% of AC cases (19). The patient in the present case was positive for GCDFP-15, which was consistent with the apocrine features within the pathomorphologically abundant eosinophilic cytoplasm. Furthermore, the IHC analysis identified that the lesion was consistent with a tumor exhibiting apocrine features. With regard to invasive carcinomas, GCDFP-15 expression has been identified as significantly lower in small tumors or tumors exhibiting lymph node metastasis (20). Previous studies have suggested that compared with invasive carcinomas, ACs are not associated with a significant difference in survival rate; however, they exhibit reduced lymph node metastasis and lymphatic invasion, and an improved prognosis and treatment response (21,22). Thus, it was suggested that the patient in the present case, may experience an improved treatment response as the tumor exhibited apocrine features (pathomorphologically and in the IHC analysis); however, tumor relapse and metastasis occurred in the patient at an early stage. The factors that contribute to tumor relapse and metastasis include a large tumor diameter, the presence of lymph node metastasis and the dominance of a high-grade SCC (2,14). Identification of histological subtypes is required for tumor grading in cases where differentiation between squamous and apocrine features is complex.

AR, in addition to GCDFP-15, is an effective IHC marker for identifying apocrine features and is frequently expressed in AC (11,18,23); in the present case, the AR was expressed in the nucleus and/or the cytoplasm of the tumor cells. Although the analysis performed was not able to explicitly distinguish between the squamous and apocrine features, the expression of AR may be significant in tumors that exhibit squamous and apocrine features. Further investigation is required to clarify this observation for pre- and post-operative adjuvant treatments.

In conclusion, although the differentiation between SCC and AC using hematoxylin and eosin staining is complex, particularly when eosinophilic cells are predominant within the tumor, performing IHC staining for markers (such as GCDFP-15 and AR) may be essential for the grading of tumors and the development of appropriate treatment.

## References

[1] Rosen PP (2009). Carcinoma with metaplasia. Rosen’s Breast Pathology.

[2] Yamaguchi R, Horii R, Maeda I (2010). Clinicopathologic study of 53 metaplastic breast carcinomas: their elements and prognostic implications. Hum Pathol.

[3] Wargotz ES, Norris HJ (1989). Metaplastic carcinomas of the breast. I Matrix-producing carcinoma. Hum Pathol.

[4] Wargotz ES, Deos PH, Norris HJ (1989). Metaplastic carcinomas of the breast. II Spindle cell carcinoma. Hum Pathol.

[5] Wargotz ES, Norris HJ (1989). Metaplastic carcinomas of the breast. III Carcinosarcoma. Cancer.

[6] Wargotz ES, Norris HJ (1990). Metaplastic carcinomas of the breast. IV Squamous cell carcinoma of ductal origin. Cancer.

[7] Wargotz ES, Norris HJ (1990). Metaplastic carcinomas of the breast: V. Metaplastic carcinoma with osteoclastic giant cells. Hum Pathol.

[8] Weigelt B, Reis-Filho JS (2009). Histological and molecular types of breast cancer: is there a unifying taxonomy?. Nat Rev Clin Oncol.

[9] Schmitt FC, Reis-Filho JS (2002). Oncogenes, granules and breast cancer: what has c-myc to do with apocrine changes?. Breast.

[10] Selim AG, Wells CA (1999). Immunohistochemical localisation of androgen receptor in apocrine metaplasia and apocrine adenosis of the breast: relation to oestrogen and progesterone receptors. J Clin Pathol.

[11] Tsutsumi Y (2012). Apocrine carcinoma as triple-negative breast cancer: novel definition of apocrine-type carcinoma as estrogen/progesterone receptor-negative and androgen receptor-positive invasive ductal carcinoma. Jpn J Clin Oncol.

[12] Japaze H, Emina J, Diaz C (2005). ‘Pure’ invasive apocrine carcinoma of the breast: a new clinicopathological entity?. Breast.

[13] Toikkanen S (1981). Primary squamous cell carcinoma of the breast. Cancer.

[14] Yamaguchi R, Tanaka M, Kondo K (2012). Immunohistochemical study of metaplastic carcinoma and central acellular carcinoma of the breast: central acellular carcinoma is related to metaplastic carcinoma. Med Mol Morphol.

[15] Frable WJ, Kay S (1968). Carcinoma of the breast. Histologic and clinical features of apocrine tumors. Cancer.

[16] Abati AD, Kimmel M, Rosen PP (1990). Apocrine mammary carcinoma. A clinicopathologic study of 72 cases. Am J Clin Pathol.

[17] Vranic S, Tawfik O, Palazzo J (2010). EGFR and HER-2/neu expression in invasive apocrine carcinoma of the breast. Mod Pathol.

[18] Gatalica Z (1997). Immunohistochemical analysis of apocrine breast lesions. Consistent over-expression of androgen receptor accompanied by the loss of estrogen and progesterone receptors in apocrine metaplasia and apocrine carcinoma in situ. Pathol Res Pract.

[19] Niemeier LA, Dabbs DJ, Beriwal S, Striebel JM, Bhargava R (2010). Androgen receptor in breast cancer: expression in estrogen receptor-positive tumors and in estrogen receptor-negative tumors with apocrine differentiation. Mod Pathol.

[20] Honma N, Takubo K, Akiyama F (2005). Expression of GCDFP-15 and AR decreases in larger or node-positive apocrine carcinomas of the breast. Histopathology.

[21] Tanaka K, Imoto S, Wada N, Sakemura N, Hasebe K (2008). Invasive apocrine carcinoma of the breast: clinicopathologic features of 57 patients. Breast J.

[22] Ogiya A, Horii R, Osako T (2010). Apocrine metaplasia of breast cancer: clinicopathological features and predicting response. Breast Cancer.

[23] Sapp M, Malik A, Hanna W (2003). Hormone receptor profile of apocrine lesions of the breast. Breast J.

